# Optical Determination of the Internal Capillary Diameter Used in Taylor Dispersion Analysis

**DOI:** 10.1002/elps.202400156

**Published:** 2024-11-28

**Authors:** Sébastien Roca, Laurent Leclercq, Philippe Gonzalez, Chutintorn Somnin, Marta Garrido Alvarez, Laura Dhellemmes, Joseph Chamieh, Hervé Cottet

**Affiliations:** ^1^ IBMM University of Montpellier, CNRS, ENSCM Montpellier France

**Keywords:** capillary diameter, capillary electrophoresis, diffusion coefficient, optical measurement, Taylor dispersion analysis

## Abstract

In this work, we describe an optical setup to determine the internal diameter of narrow bore fused silica capillary used in capillary electrophoresis and Taylor dispersion analysis (TDA). Indeed, fluctuations up to about ±3–4 µm on the capillary I.D. can generate important inaccuracy on the hydrodynamic radius determination by TDA. Calibration of the optical set‐up, impact of the operator and of the placement of the capillary in the focal plane, and the influence of the way to cut the capillary were investigated and discussed. This optical set‐up was next used to determine capillary I.D. on a 60 m long capillary spool. Relatively small variations were observed along a 60 m capillary spool (0.3 µm maximum variation), while important I.D. fluctuations can be observed from capillary batch to batch. Taking three capillaries of three different nominal I.D. values, *R*_h_ values of sodium benzoate obtained by TDA were not significantly different if the capillary I.D. were optically measured, while significant variations were observed with the nominal I.D. values. A protocol based on TDA of sodium benzoate was proposed for calibrating narrow bore fused silica capillary I.D. without the use of optical measurements for researchers that would not have access to such optical equipment.

**Abbreviations**
BGE: background electrolyte
CE: capillary electrophoresis
*D*
: diffusion coefficient
I.D.: internal diameter
NaOH: sodium hydroxide
OT‐LC: open‐tubular liquid chromatography
*R*_h_
: hydrodynamic radius
TDA: Taylor dispersion analysis

## 1 Introduction

The use of thin fused silica capillaries for analytic purposes such as capillary electrophoresis (CE) [1], Taylor dispersion analysis (TDA) [2, 3], or open‐tubular liquid chromatography (OT‐LC) [4, 5] allowed full automation and low sample and eluent consumptions. One of the major issues with the use of capillaries is the knowledge of their exact internal diameter (I.D.) value. When ordering a capillary spool, the operator can select the desired inner diameter (usually 50 µm I.D., with most of the applications in the range of 100–25 µm I.D., for classical CE or TDA uses). This I.D. value is hereafter referred to as the nominal value. Due to slight variabilities during capillary production, differences between the nominal I.D. and the actual value can exist in a given tolerance range given by the capillary manufacturer. For that reason, each capillary spool is generally provided with the actual capillary I.D. for the beginning and the end of the spool. Accordingly, the specifications of the provider include a tolerance interval that depends on the nominal I.D. value (25 ± 2 µm; 50 ± 3 µm; 75 ± 3 µm; 100 ± 4 µm for the TSP Polymicro polyimide coated fused silica capillary tubings) [6]. A more accurate I.D. specification is also available from Molex Polymicro (Tight‐Tolerance capillaries, 25 ± 1 µm; 50 ± 1 µm; 75 ± 2 µm; 100 ± 2 µm) [7] at higher price. Within this tolerance interval, fluctuations on the actual capillary I.D. can be obviously observed and contribute to lowering the capillary‐to‐capillary intermediate precision in CE and in TDA. Indeed, in CE, the joule heating is directly proportional to the capillary internal surface, which scales as the capillary radius to the power 2. Fluctuations in the capillary radius change the dissipated power, and therefore, the equilibrated temperature, and by consequence, the viscosity of the electrolyte and the electrophoretic mobility, and finally, the solute migration times. In TDA, apart from the elution time that is affected by the capillary I.D. via the Poiseuille law, the determination of the diffusion coefficient (*D*) according to Taylor's equation is strongly influenced by the capillary I.D. since *D* scales with the capillary radius to the power 2 according to [8, 9, 10, 11, 12, 13]:

(1) $$D=\frac{d_{c}^{2}t_{0}}{96\;\sigma^{2}}$$

with *d*_c_ being the capillary I.D. value (in m), *t_0_* the average elution time (in s), and *σ^2^* the temporal variance of the elution profile (in s^2^).

Early experiments relative to glass capillary and capillary I.D. variations were performed in 1909 [14]. The length of a thin mercury thread within the tubing was determined by moving it along the capillary with air pressure. Changes in the cross‐sectional area were observed by tracking the changes in the length of the mercury thread. More recently, Kwon et al. also used x‐ray to estimate glass capillary I.D. [15]. The x‐ray beam goes through the glass capillary before being detected by a complementary metal‐oxide semiconductor (CMOS) sensor. The I.D. was measured and analyzed by the pixel values of the x‐ray image. Thus, accuracy of such methods depends on the pixel size (here, 0.33 µm/pixel), and an average internal diameter of 108.33 ± 0.67 µm was measured. Another method to measure the internal diameter of narrow glass syringe tubes or capillaries (with a typical I.D. of around 0.5 mm) is based on gravimetry. The tube is filled with a known liquid, the average I.D. being calculated by the comparison between the masses of the empty tube and the filled one [16, 17]. This method was used for the determination of capillary I.D. going from 0.5 up to 1.5 mm but is not adapted for narrow bore capillaries used in CE or TDA due to low capillary volumes. It is also possible to use the Hagen‐Poiseuille law using a liquid of known viscosity (e.g., pure water) at a given temperature, and to determine the capillary I.D. based on the residence time of a marker under controlled pressure [18]. However, this method is based on the assumptions that the applied pressure and the capillary temperature are perfectly controlled, which is not a priori obvious. Another protocol consisted in frontal optical measurement of the I.D. using high resolution camera. Molex Polymicro used an Olympus PME3 inverted microscope fitted with a cross hair filar and a Microcode II digital encoder to measure actual I.D. used in GC (250‐530 µm I.D.) [19]. Using such instrumentation, they could verify that the tolerance interval of these GC capillaries was decreased from ± 12 µm in 1990 down to ± 6 µm in 2001 [19]. Later on, they applied a similar optical setup and demonstrated that they could improve the process of production on a 250 µm I.D. capillary by lowering the standard deviation on the internal diameter from 1.62 µm in 2004 down to 1.29 µm in 2011 [20]. Macomber et al. [21] also investigated the measurements of capillary I.D. on a small‐bore capillary tubing using a Pinnacle Vision system including an optical system outfitted with a 20× lens and calibrated using a circular target. They verified the specifications of TSP020375 capillary (i.e. I.D. 20 ± 2 µm) by optically measuring capillary I.D. each 10 m on four lots of 110 m capillaries. The I.D. variations with each lot was comprised between 0.4 and 1.2 µm.

Following the typical ± 3 µm tolerance for the 50 µm I.D. capillary, inter‐capillary comparisons may lead to a maximal relative error of 24 % on the measurement of *D* by TDA according to Equation (1). This maximal error is lowered to 8% in the case of tight tolerance capillaries (50 ± 1 µm). Regardless of the tolerance and potential variations between batches of capillaries, or even within a single capillary spool, it is important for researchers to have the ability to precisely measure the capillary I.D.

In this work, we describe an optical setup that can be used to determine the internal diameter of fused silica capillary by frontal measurement (i.e., by placing the capillary end in front of the objective of a camera) using an optical bench. Calibration of the set‐up, impact of the operator and of the placement of the capillary in the focal plane, and the influence of the way to cut the capillary were investigated and discussed. This set‐up was next used to determine the capillary I.D. on a 60 m long capillary spool. Finally, a protocol based on TDA of sodium benzoate was proposed for calibrating narrow bore fused silica capillaries without the use of optical measurements for researchers that would not have access to such optical equipment.

## 2 Materials and Methods

### 2.1 Chemicals

Polymicro Technology (Molex, Phoenix, AZ, USA) supplied 25, 50 and 75 µm I.D. (nominal values) capillaries under the TSPXXX375 designation where, XXX is the I.D. in µm. Sodium tetraborate decahydrate (Borax, ≥ 99.5% purity) was purchased from Sigma‐Aldrich (Saint‐Quentin‐Fallavier, France). Sodium hydroxide (NaOH, 98 % purity) was purchased from Fluka (Saint‐Quentin‐Fallavier, France). Sodium benzoate (99 % purity) was purchased from Merck (Saint‐Quentin‐Fallavier, France). All aqueous solutions were prepared using deionized water (18 MΩ) delivered by a Synergy UV water purification system (Millipore, Fontenay sous Bois, France).

### 2.2 Optical Bench

**FIGURE 1:**
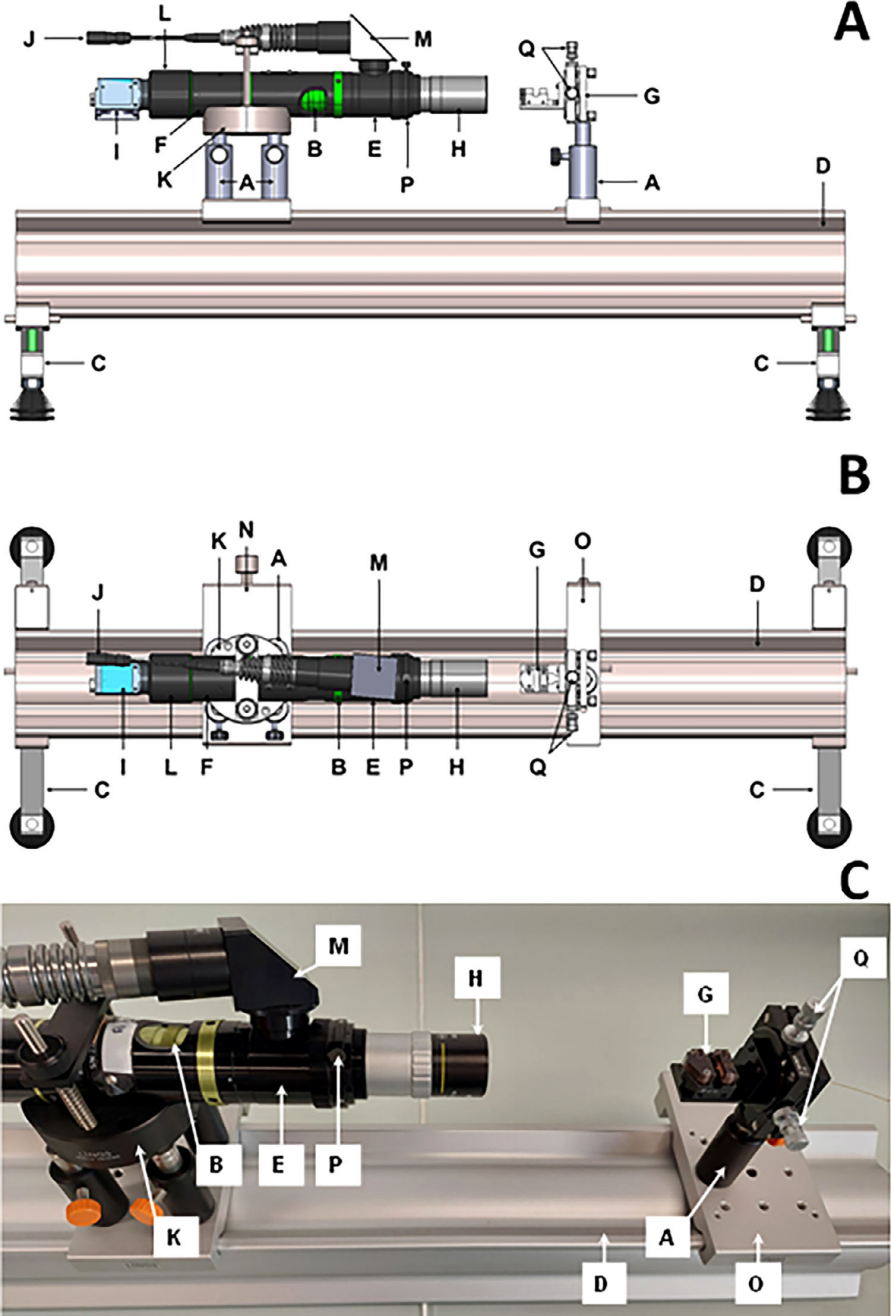
Visualization of the optical bench system used in this work from side view (A), top view (B), and picture of the whole setup (C). All letters correspond to the abbreviations given in Table 1.

Optical bench components and software were purchased or provided from Photonlines (Pace, France) and assembled in the laboratory. A 3D representation of the optical bench is displayed in Figure 1 (side view Figure 1A, top view Figure 1B). Name, abbreviation, and quantity of each component are given in Table 1. The sizing of the capillary ID was performed based on a picture of the capillary I.D. via the optical setup and using IC measure software. The overall price of the listed components was about 9 000 euros in 2023.

**TABLE 1:** List of the components used to build the optical bench.

Abbreviation	Name	Quantity
**A**	Post base + Optical post 14 × 40; M6/M4	5
**B**	Manual zoom	1
**C**	Double leg	2
**D**	Profile X 95; 640 mm	1
**E**	Coax illuminator 5 mm focus manual	1
**F**	Camera tube 0.4	1
**G**	Capillary holder	2
**H**	Optem 10X M Plan APO Objective	1
**I**	Camera (DMK 33UX226)	1
**J**	Led coax illuminator	1
**K**	V‐Clamp Holder 50 ‐ M	2
**L**	C/CS Camera Mount	1
**M**	Coax Right Angle	1
**N**	Carrier X 95‐80, colorless, w/ hole pattern	2
**O**	Carrier Positioner 50 X95‐30	1
**P**	Focal adjustment	1
**Q**	Adjusting screws	1
/	Positive NBS 1963A Resolution Target	1
/	Target carrier	1
/	Set of screws M6x16 (50 pcs) and M4x16	1

### 2.3 Sample Preparation

Sodium benzoate solution was prepared at the concentration of 1 g/L (6.9 × 10^−3^ mol/L) in 50 mM sodium borate buffer, pH 9.2. The 50 mM sodium borate buffer was prepared by dissolving 477.5 mg of borax (sodium tetraborate decahydrate, molecular mass 382 g/mol) in 100 mL of water.

### 2.4 Taylor Dispersion Analysis

TDA experiment was performed on a Beckman PACE MDQ system (Beckman Coulter, Villebon‐sur‐Yvette, France), piloted by 32 Karat software. Fused silica capillaries (50 cm total length, 41.5 cm to the UV detection window) were conditioned by flushing 1 M NaOH solution (5 min, 1 bar) followed by pure water (5 min, 1 bar). TDA was performed in a 50 mM sodium borate buffer at pH 9.2. The capillary temperature was set at 25°C. The injection parameters (pressure and time) and the mobilization pressures were calculated to inject exactly the same plug length of sodium benzoate solution for all capillary I.D. values while verifying $Pe=\frac{ld_{c}}{2t_{0}D}\geq 40$ and $\tau =\frac{4t_{0}D}{d_{c}^{2}}\geq 1.25$ conditions of validity of TDA [22], where *l* is the capillary length to the detector. Namely, sodium benzoate was injected for 5.2 s at 1.0 psi for 25 µm I.D. capillary; for 3.2 s at 0.4 psi for 50 µm I.D. capillary, and for 3.0 s at 0.2 psi for 75 µm I.D. capillary. Sodium benzoate was mobilized at 5.8 psi for 25 µm I.D. capillary; at 1.4 psi for 50 µm I.D. capillary, and at 0.6 psi for 75 µm I.D. capillary.

## 3 Results and Discussion

Measuring the capillary I.D. requires firstly the calibration of the optical setup presented in Section 2.2.

### 3.1 Optical Bench Calibration

**FIGURE 2:**
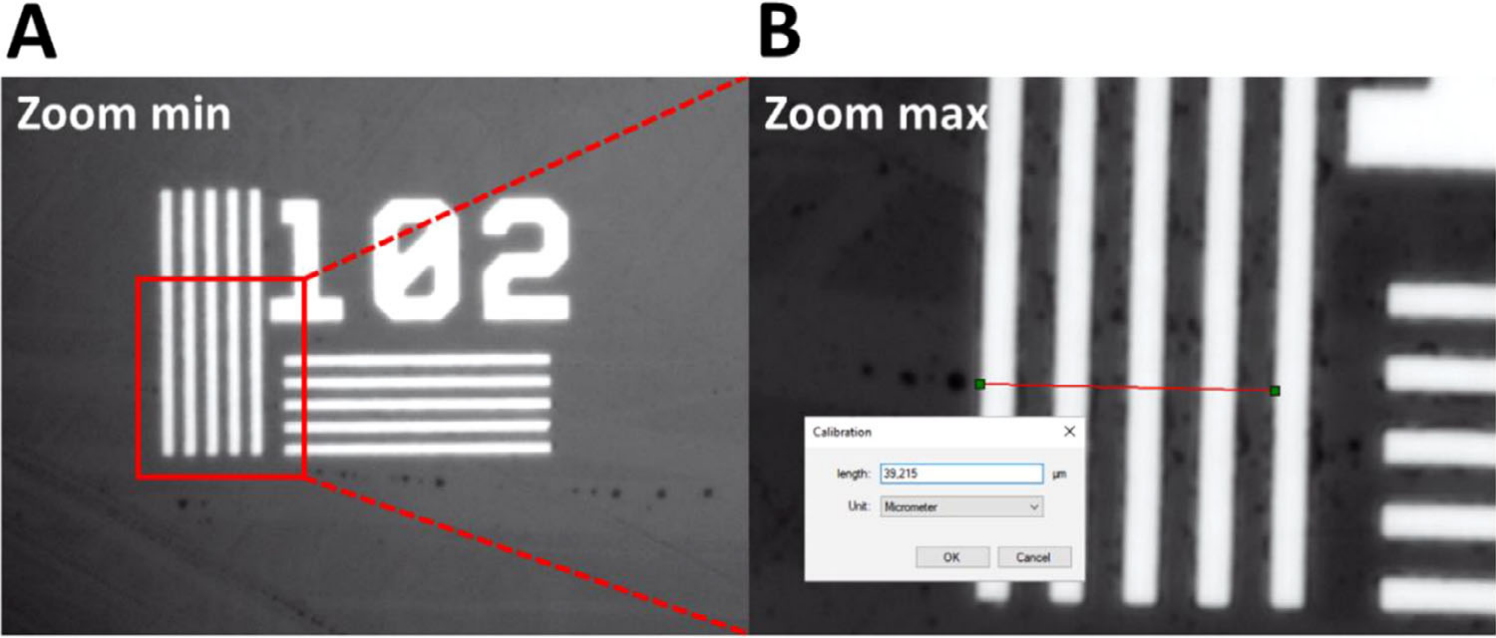
Visualization of *Positive NBS 1963A Resolution Target* observed at minimum zoom (A) and maximum zoom (B) for optical bench calibration. Calibration was performed by entering into the IC Measure software the distance between four white lines (4 × 9.8 = 39.2 µm) from edge to edge as depicted by the red line in (B).

The aim of the calibration of the optical setup is to determine the distance between two pixels displayed by IC Measure software on the computer screen, for a given position of the zoom (see part B of the setup in Figure 1) selected by the operator. To achieve this, a *Positive NBS 1963A Resolution Target* was used. This target features lines regularly spaced by a known, calibrated distance. The number indicated on the target (Figure 2A) corresponds to the number of lines for 1 mm. In the example given in Figure 2A, there are 102 lines per millimeter which means that the exact distance between the left edge of the first white line and the left edge of the second white line is 1000/102 = 9.8 µm. In this study, all measurements were taken at maximum zoom, and calibration was performed by entering the distance corresponding to 4 lines (4 × 9.8 = 39.2 µm, see Figure 2B).

To measure the capillary I.D., as the depth of field is shallow on this optical bench, the zoom was first used in the minimum position to place the capillary in the focus plane (to visualize the capillary with a sharp image). The zoom was then switched to maximum position for better measurement precision. To ensure that the calibration did not move once the zoom was switched (from max / min / max positions), the measurement of distance between the four lines of the target was repeated 4 times, with results displayed in Table 2. ANOVA test (95% confidence) shows no significant variation (average value obtained over 12 measurements: 39.26 ± 0.06 µm). The calibration is therefore robust, and the various manipulations to place the capillary cross‐section in the focus plan did not influence the optical measurement.

**TABLE 2:** Study of the impact of zoom removal and re‐positioning of the *Positive NBS 1963A Resolution Target* on the optical bench regarding the measurement of the distance between four white lines on the target. Each measurement represents a mouse trace of the distance between four lines on the software (test pattern position 102, *n* = 4).

Repetition	Measurement 1 (µm)	Measurement 2 (µm)	Measurement 3 (µm)
# 1	39.32	39.28	39.24
# 2	39.18	39.22	39.31
# 3	39.23	39.28	39.27
# 4	39.26	39.30	39.22

### 3.2 Capillary Internal Diameter Measurement: Impact of Operator, Calibration, and Capillary Placement

#### 3.2.1 Internal Diameter Measurement

**FIGURE 3:**
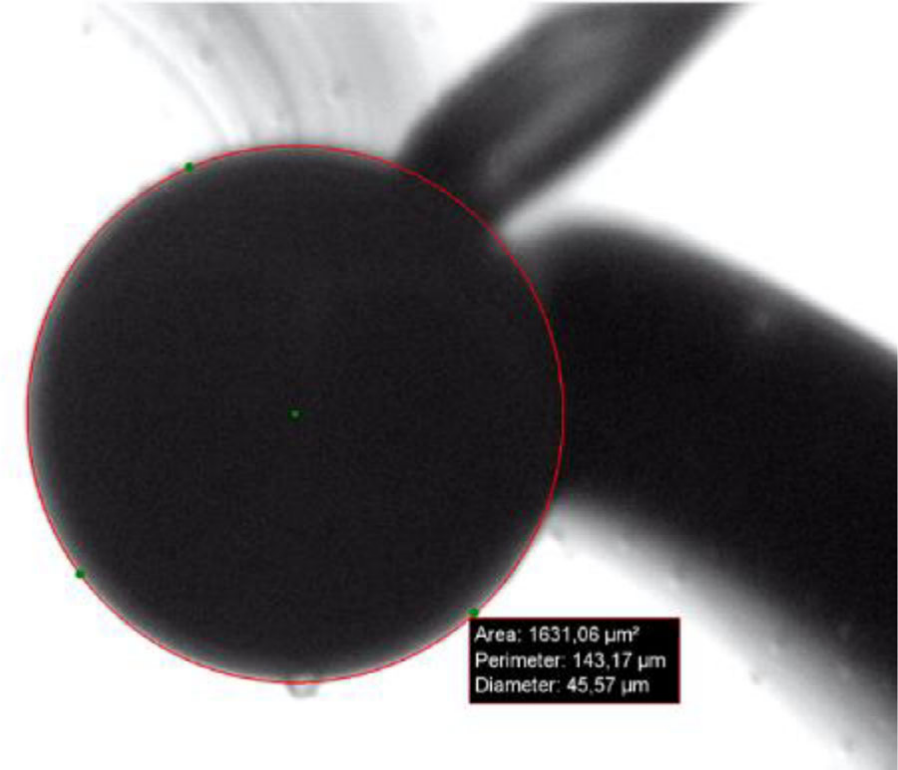
Zoom on a 50 µm nominal I.D. fused silica capillary as obtained by the optical setup at maximal zoom position. Measurement of the capillary I.D. was obtained using IC Measure software. Measured I.D.: 45.6 µm (spool I.D. as given by the provider: 47.0 µm).

Once the optical bench was calibrated, the capillary I.D. was measured by placing it in the capillary holder (see letter **G** in Figure 1), before adjusting it to the focus plane with the manual zoom (letter **B** in Figure 1) on the minimum position. Once the capillary could be sharply observed on the minimal zoom position, a zoom‐into maximum position was performed (letter **B** in Figure 1), while adjusting the focal length (letter **P** in Figure 1). To place the internal diameter in the center of the image, micrometric adjustments were made using the screws on the support (see letter **Q** in Figure 1). When the internal diameter is at the center of the image, the I.D. was measured by visually placing 3 points on the contour (green dots and red circle, see Figure 3). IC Measure software displayed the value of the capillary diameter based on the circle previously drawn (45.57 µm in the present case, see Figure 3).

#### 3.2.2 Operator Impact for a Previously Calibrated Optical Bench and a Capillary Placed in the Focal Plane

After optically calibrating the measurement system, the I.D. of the same capillary (nominal value of 50 µm) was measured by four different operators without changing the focus or positioning of the capillary. This was performed to judge the impact of visual adjustment of the capillary I.D. by the red circle in the IC Measure software. On average, the I.D. was 45.54 µm with standard deviation of 0.12 µm (0.26 % RSD). All measurements are presented in Table 3. Repeatability was found excellent. The measured value compared with the value announced by the manufacturer (47.0 µm) showed a relative bias of −3.1%. An ANOVA test on this series of data confirmed that the operator factor has no significant influence on the result at 95% confidence level, validating the visual optical determination of diameter by different operators (detailed ANOVA is presented in Table S1).

**TABLE 3:** Repeated measurement of capillary internal diameter (*n* = 10) by four different operators without capillary removal between measurements and operators.

Measurement #	Operator 1	Operator 2	Operator 3	Operator 4
1	45.57	46.00	45.36	45.61
2	45.68	45.63	45.45	45.74
3	45.61	45.51	45.54	45.50
4	45.39	45.32	45.56	45.57
5	45.46	45.70	45.46	45.64
6	45.44	45.48	45.36	45.48
7	45.28	45.53	45.48	45.61
8	45.52	45.42	45.53	45.59
9	45.52	45.67	45.46	45.59
10	45.65	45.64	45.54	45.50
Average (µm)	45.51	45.59	45.47	45.58
Standard derivation (µm)	0.12	0.19	0.07	0.08
RSD (%)	0.27	0.41	0.16	0.17

#### 3.2.3 Operator Impact for a Calibrated Optical Bench and a Capillary Not Initially Placed in the Focusing Plane

Ten successive internal diameter measurements were carried out by 4 different operators, by systematically removing and replacing the capillary between 2 measurements. This was performed to evaluate the impact of the choice of focusing plane (image sharpness) and the positioning of the capillary in relation to the optical axis. All the measurements are gathered in Table 4, and the ANOVA test is shown in Table S2. It was demonstrated that the operator has no influence, even taking the placement of the capillary and the adjustment of the optical system into account at 3% confidence level. Taking 5% confidence level, the placement of the capillary by the operator has a significant impact but with relatively low differences between operators (≤ 0.2 µm). The average value of the internal diameter obtained for this set of experiments was 45.85 ± 0.24 µm, with very low RSD of 0.37%.

**TABLE 4:** Repeated measurement of capillary internal diameter (*n* = 10) by four different operators, with capillary removal between each measurement.

Measurement #	Operator 1	Operator 2	Operator 3	Operator 4
1	46.17	45.95	45.73	45.52
2	45.55	45.98	45.88	45.85
3	45.92	46.03	45.91	45.84
4	45.92	45.9	46.04	45.71
5	46.22	45.71	45.86	45.86
6	45.8	45.95	45.93	45.81
7	45.81	45.75	46.12	45.81
8	46.29	45.75	45.86	45.75
9	45.65	45.84	46.01	45.68
10	45.96	45.85	46.18	45.64
Average (µm)	45.93	45.87	45.95	45.75
Standard derivation (µm)	0.24	0.11	0.14	0.11
RSD (%)	0.53	0.24	0.29	0.24

#### 3.2.4 Impact of Calibration Procedure on Measurement

The impact of the calibration procedure on the I.D. determination was studied by making three series of three measurements obtained after a new calibration (see procedure in section 3.1). The results of this set of experiments are presented in Table 5. An average of 45.53 µm (RSD of 0.11%) was found, ruling out any influence of calibration on the measurement.

**TABLE 5:** Measurement of capillary internal diameter (*n* = 3) for new calibrations of the optical bench.

	Calibration		
Measurements #	1	2	3
1	45.47	45.41	45.53
2	45.45	45.65	45.6
3	45.61	45.46	45.61
Average (µm)	45.51	45.51	45.58
Standard derivation (µm)	0.09	0.13	0.04
RSD (%)	0.19	0.28	0.1

### 3.3 Tracking Internal Diameter Fluctuations on a Capillary Spool and Influence of the Cutting Protocol

**FIGURE 4:**
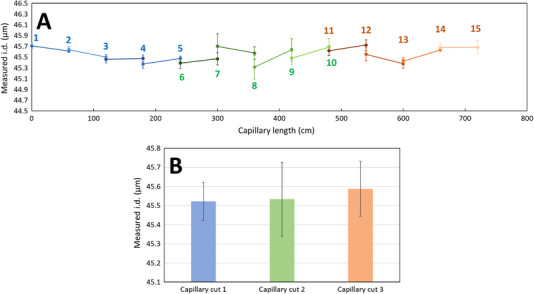
Measurement of the internal diameter of a 7.2 m long 50 µm I.D. capillary every 60 cm at both capillary ends (*n* = 3 measures for each end) (A) and impact of the cutting procedure on the I.D. values and standard deviations (B). The first firve cuts (data in blue) were made using the regular cut, cuts from 6 to 10 were made using the twisted cut (data in green), and the last five cuts were made using the circular cut (data in orange) according to protocols presented in Figure S1. Error bars are ± 1 SD. Capillary I.D. values given by Polymicro on the spool: 47.0 µm begin, 47.2 µm end.

After confirming the robustness of the optical measurement regarding the change in the operator and the calibration protocol, 3 cutting protocols were compared. This was performed to estimate the potential impact of the cutting procedure, and therefore the regularity of the capillary surface in the cut section on the measurement of the I.D. value. The measurement of the capillary I.D. was monitored over a 7.2 m section of a nominal 50 µm I.D. capillary (capillary I.D. values given by Polymicro on the spool: 47.0 µm begin, 47.2 µm end) by cutting the capillary every 60 cm according to the 3 cutting modes presented in Figure S1 and hereafter referred as regular cut, twisted cut, and circular cut. Cutting 1 (regular cut) gave the best fit between two successive capillary ends (see blue points in Figure 4A), with the lowest standard deviations (Figure 4B). This cutting mode is recommended and corresponds to the intuitive way to cut the capillary. Regarding the twisted cut method (green histogram) and the circular cut (red histogram) in Figure 4B, higher standard derivations were calculated in the I.D. measurement.

**FIGURE 5:**
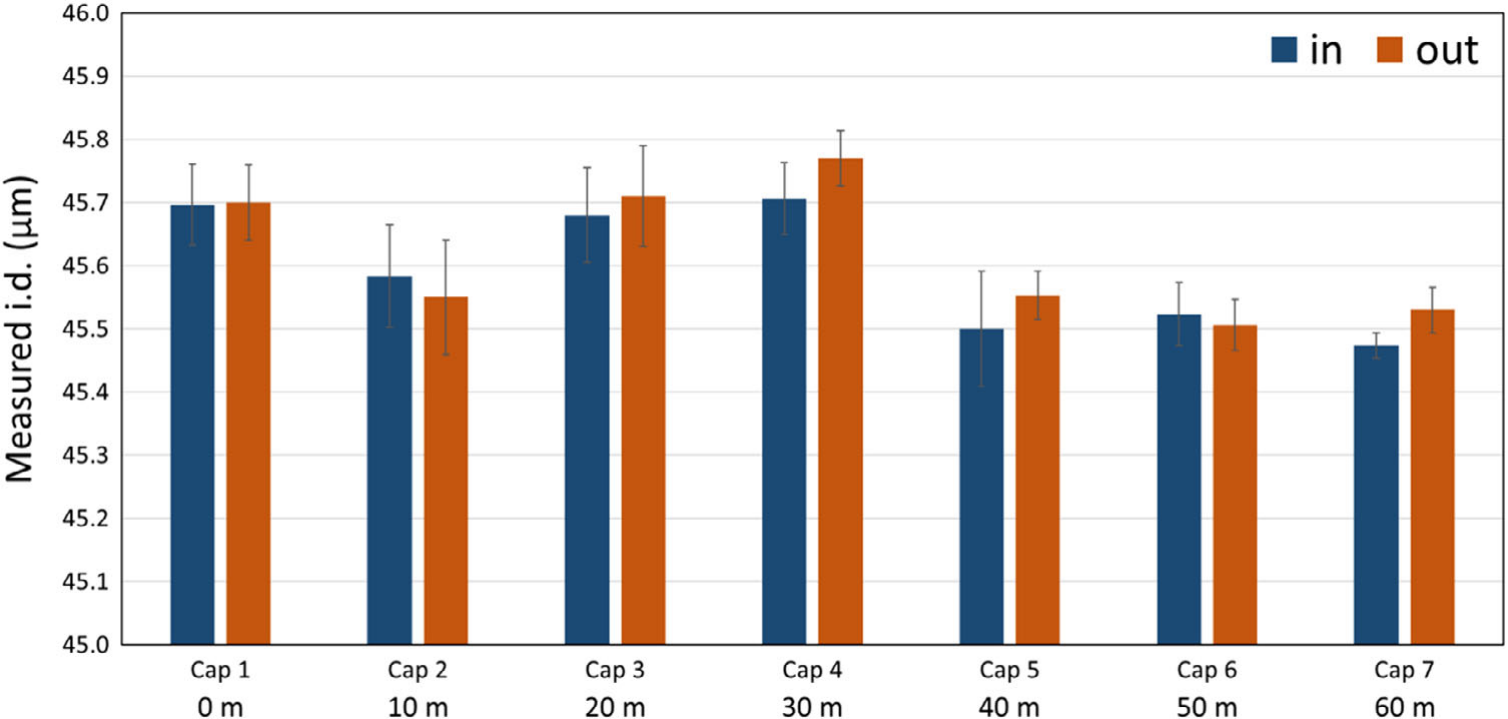
Optical measurement (*n* = 3) of the capillary I.D. (nominal value 50 µm) over 60 m for a given single capillary spool. Error bars are ± 1 SD. Capillary I.D. values given by Polymicro on the spool: 47.0 µm begin, 47.2 µm end.

A second series of I.D. measurements was carried out on a 60 m capillary spool (same spool as in Figure 4). This series was performed by cutting 10 cm of capillary every 10 m (regular cut) and measuring the I.D. at the inlet and outlet of the 10 cm long capillary. A total of 14 measurements were performed, and the results are shown in Figure 5. It was observed that the capillary inlet and outlet values were almost identical for each measurement, which is consistent with very low I.D. fluctuations over a 10 cm length. On average, over the 60 m capillary length, the I.D. was 45.61 µm (± 0.10 µm SD, 0.22% RSD), which is similar to that previously measured on the same spool. Maximum variations in I.D. in the order of 0.3 µm were observed along the 60 m capillary spool (i.e. about 0.64% fluctuations) in agreement with typical values obtained by the manufacturer on 20 µm I.D. capillaries [21].

Higher variation was observed between the value announced on the spool by the manufacturer (47.0 µm I.D.) and the one measured optically (45.6 µm; ΔI.D. = −1.45 µm corresponding to −3.1%) but still within the announced tolerance range of ± 2 µm compared to the announced value (but not compared to the 50 µm nominal value). In conclusion, it is preferable to measure the capillary I.D. before doing TDA experiments for better inter‐capillary intermediate precision and better TDA sizing accuracy. In view of the small variations present along the capillary spool, a single measurement for a given spool could be considered as sufficient for a correct estimation of the spool average capillary I.D.

### 3.4 Impact of Capillary I.D. on TDA Measurement and I.D. Determination Using TDA

To experimentally investigate the impact of the capillary I.D. on TDA experiments and on *R*_h_ and *D* measurements, three fused silica capillaries of different nominal I.D. (75, 50, and 25 µm) were used. All capillaries were 50 cm long (effective length of 41.5 cm) and were used to analyze sodium benzoate at 1 g/L in 50 mM borate buffer (pH 9.2) using UV detection at 200 nm. Injection parameters and mobilization pressures were adjusted to verify the conditions of validity of TDA (as described in Section 2.4). In the case of 25 µm capillary I.D., *D* and *R*_h_ were calculated using the full Taylor‐Aris expression [6, 7, 8] since the contribution of axial molecular diffusion is not completely negligible. The capillary I.D. were measured using the optical bench, following the protocols described in Sections 3.1 (calibration) and 3.2. (measurement). The inlet and the outlet capillary I.D. were measured three times each and the average value was used for the TDA experiment (Table 6).

**TABLE 6:** Measurement of the actual capillary I.D. (*n* = 3) of 25, 50, and 75 µm nominal values with a calibrated optical bench. The manufacturer value corresponds to the average values given for the inlet and outlet ends of the spool.

	Nominal I.D. (µm)	75	50	25
	Manufacturer I.D.^a^ (µm)	75.49	47.10	25.20
Inlet	Measurement 1	73.65	45.37	25.92
	Measurement 2	74.32	45.33	26.08
	Measurement 3	73.8	45.4	25.96
Outlet	Measurement 1	73.65	45.77	25.82
Outlet	Measurement 2	73.77	45.83	25.92
Outlet	Measurement 3	73.74	45.71	25.95
	Average optical I.D. (µm)	73.82	45.57	25.94
	Standard derivation (µm)	0.25	0.23	0.09
	RSD (%)	0.34	0.49	0.36

^a^ Corresponding to the average inlet and outlet I.D. values given by the manufacturer.

**FIGURE 6:**
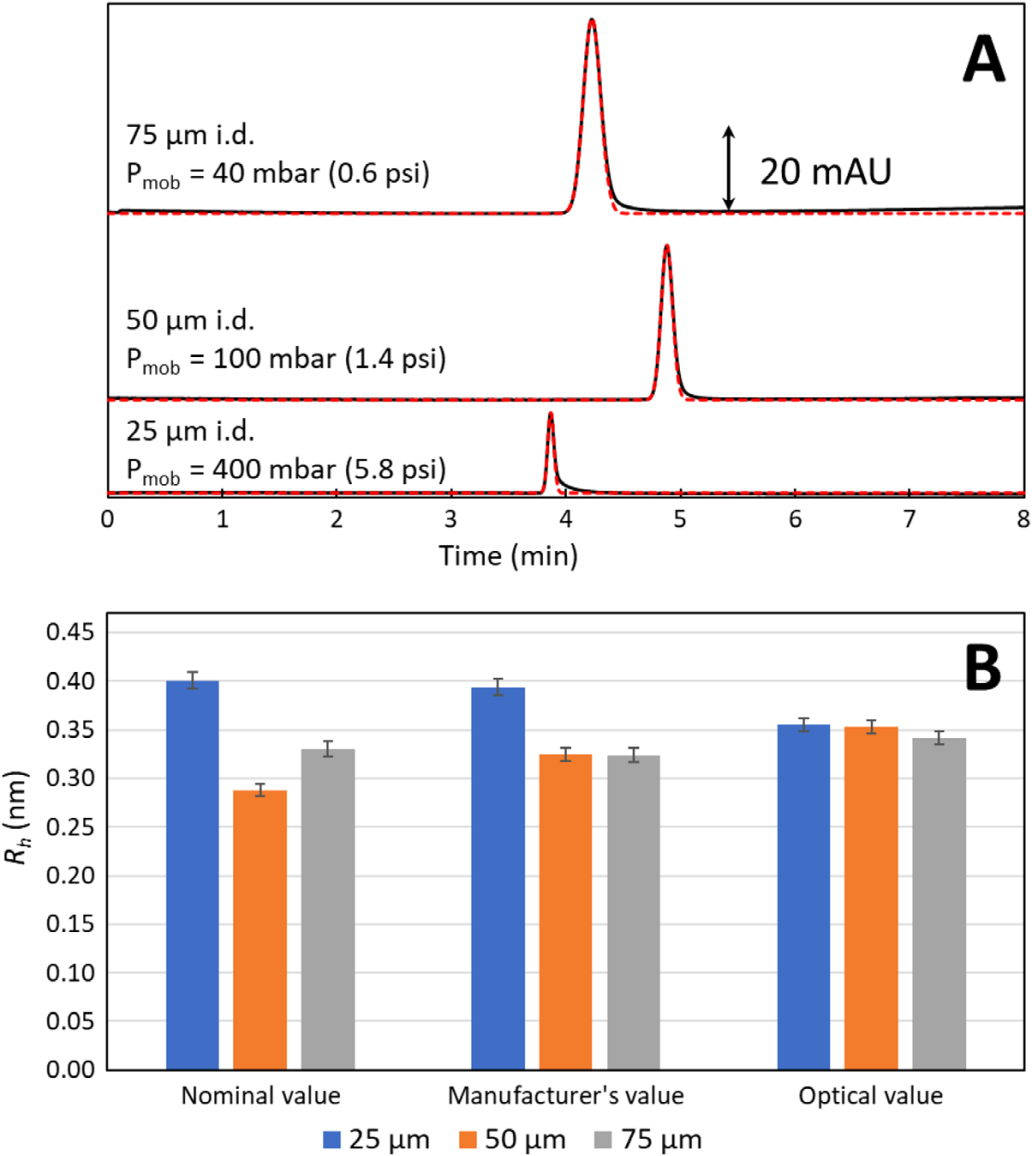
TDA (*n* = 5) of sodium benzoate in borate buffer with three different capillary I.D. (A) and impact of *d*_c_ value on *R*_h_ measurement (B). Experimental conditions: fused silica capillary, 50 cm total length (41.5 cm to detector) × 75, 50 or 25 µm nominal I.D. Eluent: 50 mM borate buffer (pH 9.2). Hydrodynamic injection parameters: see Section 2.4. Solute: sodium benzoate at 1 g/L in the eluent. Mobilization pressure as indicated in the figure. Temperature: 25°C. UV detection: 200 nm. Viscosity of the eluent used for *R*_h_ calculation: 0.89 × 10^−3^ Pa s at 25°C. Error bars are ± 1 SD.

As observed in Figure 6A, TDA experiments gave Gaussian elution profiles for the three capillary I.D. Gaussian fitting was used to determine the temporal variance of the elution profile (see red dashed line in Figure 6A). *D* and *R*_h_ values were determined using Equation (1) (except for 25 µm I.D. where Taylor–Aris expression was used, see equation 9 in ref. [23]) and Stokes–Einstein equation. Using the nominal I.D. values, three distinct *R*_h_ were obtained for the three experiments (see Figure 6B). This can be explained by the fact that the nominal value of capillary I.D. is rarely the same value as the actual one. When changing I.D. value from the nominal one to the one given by the manufacturer, measurements performed with 50 and 75 µm capillary I.D. gave similar results. All *R*_h_ values were almost identical when using the optical I.D. value. Finally, sodium benzoate has an *R*_h_ = 0.349 ± 0.007 nm in size (*D* = 7.03 ± 0.14 × 10^−10^ m^2^/s) with no significant influence of the capillary I.D. on *R*_h_ values (ANOVA, 95% confidence level) at 25°C. These *R*_h_ and *D* values are in good agreement with the literature [24, 25, 26].

This experiment demonstrates the importance of knowing accurately the capillary I.D. value to accurately determine *D* and *R*_h_ values in TDA. This is in agreement with what we observed experimentally as TDA users when comparing TDA results obtained from different lots of capillaries. Reversely, for researchers doing TDA that do not have optical setup for precise I.D. measurement, we propose to use sodium benzoate as a calibration solute for measuring capillary I.D. using Equation (1) with *d*_c_ as the unknown parameter and *D* as 7.03 × 10^−10^ m^2^/s. For better precision, the temperature should be rigorously set at 25°C for the capillary cartridge, and if possible for the eluent vials. For instance, the use of an external circulating bath on Agilent 7100 CE equipment is advised.

## 4 Concluding Remarks

In this work, we propose the use of an optical setup to measure precisely and accurately the capillary I.D. of narrow bore fused silica capillaries used in CE and TDA. It was demonstrated that the measured internal diameter of a 50 µm I.D. fused silica capillary can vary from its nominal value (up to about ± 4 to 5 µm differences in this study) with important consequences on the accuracy of the *R*_h_ determination by TDA. The robustness of the calibration protocol, the impact of the operator and the impact of the capillary placement were studied and demonstrated. Relatively small variations were observed along a 60 m capillary spool (0.3 µm maximum variation). Taking three capillaries of three different nominal I.D. values, *R*_h_ values of sodium benzoate obtained by TDA were not significantly different if we use the capillary I.D. obtained optically, while significant variations were observed with the nominal values. These results are agreement with a squared dependence of *D* (with *d*_c_).

## Conflicts of Interest

The authors declare no conflicts of interest.

## Supporting information



Supporting Information

## Data Availability

The data that support the findings of this study are available from the corresponding author upon reasonable request.
