# Supplementary material for: Optical Determination of the Internal Capillary Diameter Used in Taylor Dispersion Analysis
Source: Electrophoresis. 2024 Nov 28;46(13-14):810–9. doi: 10.1002/elps.202400156 (PMC12449748; doi:10.1002/elps.202400156)
Supplement: Supplementary file 1 — Supporting Information [file ELPS-46--s001.docx]

Supporting information

Optical determination of the internal capillary diameter used in Taylor dispersion analysis.

Sébastien Roca, Laurent Leclercq, Philippe Gonzalez, Chutintorn Somnin, Marta Garrido Alvarez, Laura Dhellemmes, Joseph Chamieh and Hervé Cottet*

IBMM, University of Montpellier, CNRS, ENSCM, France

***Correspondence**: Prof. Hervé Cottet, IBMM, University of Montpellier, ENSCM, 34060 Montpellier, France, herve.cottet@umontpellier.fr

**Table of content:**

**Table S1.** 1-way ANOVA test (at 95% confidence level) on I.D. measurements (*n* = 10) obtained for different operators without modifying capillary placement on the optical bench…………………………………………………………………………………2

**Table S2.** 1-way ANOVA test (at 95% confidence level) on I.D. measurements (*n* = 10) obtained for different operators with systematic change of capillary placement on the optical bench between measurements. ………………………………………………2

**Figure S1.** Study of the impact of the cutting method of a fused silica capillary; regular cut (**A**), twisted break (**B**) and circular cut (**C**). ……………………………………………3

**Table S1.** 1-way ANOVA test (at 95% confidence level) on I.D. measurements (*n* = 10) obtained for different operators without modifying capillary placement on the optical bench.

|  |  |  |  |  |  |  |
| --- | --- | --- | --- | --- | --- | --- |
| *Groups* | *Count* | *Sum* | *Average* | *Variance* |  |  |
| Operator 1 | 10 | 455.12 | 45.51 | 0.015 |  |  |
| Operator 2 | 10 | 455.90 | 45.59 | 0.035 |  |  |
| Operator 3 | 10 | 454.74 | 45.47 | 0.0051 |  |  |
| Operator 4 | 10 | 455.83 | 45.58 | 0.0060 |  |  |
|  |  |  |  |  |  |  |
| ANOVA |  |  |  |  |  |  |
| *Source of variation* | *SS* | *df* | *MS* | *F* | *P-value* | *F crit* |
| Between Groups | 0.094 | 3 | 0.031 | 2.06 | 0.12 | 2.87 |
| Within Groups | 0.55 | 36 | 0.015 |  |  |  |
|  |  |  |  |  |  |  |
| Total | 0.65 | 39 |  |  |  |  |

**Table S2.** 1-way ANOVA test (at 95% confidence level) on I.D. measurements (*n* = 10) obtained for different operators with systematic change of capillary placement on the optical bench between measurements.

|  |  |  |  |  |  |  |
| --- | --- | --- | --- | --- | --- | --- |
| *Groups* | *Count* | *Sum* | *Average* | *Variance* |  |  |
| Operator 1 | 10 | 459.29 | 45.93 | 0,058 |  |  |
| Operator 2 | 10 | 458.71 | 45.87 | 0,012 |  |  |
| Operator 3 | 10 | 459.52 | 45.95 | 0,018 |  |  |
| Operator 4 | 10 | 457.47 | 45.75 | 0,012 |  |  |
|  |  |  |  |  |  |  |
| ANOVA |  |  |  |  |  |  |
| *Source of variation* | *SS* | *df* | *MS* | *F* | *P-value* | *F crit* |
| Between Groups | 0.25 | 3 | 0.084 | 3.34 | 0.030 | 2.87 |
| Within Groups | 0.91 | 36 | 0.025 |  |  |  |
|  |  |  |  |  |  |  |
| Total | 1.16 | 39 |  |  |  |  |


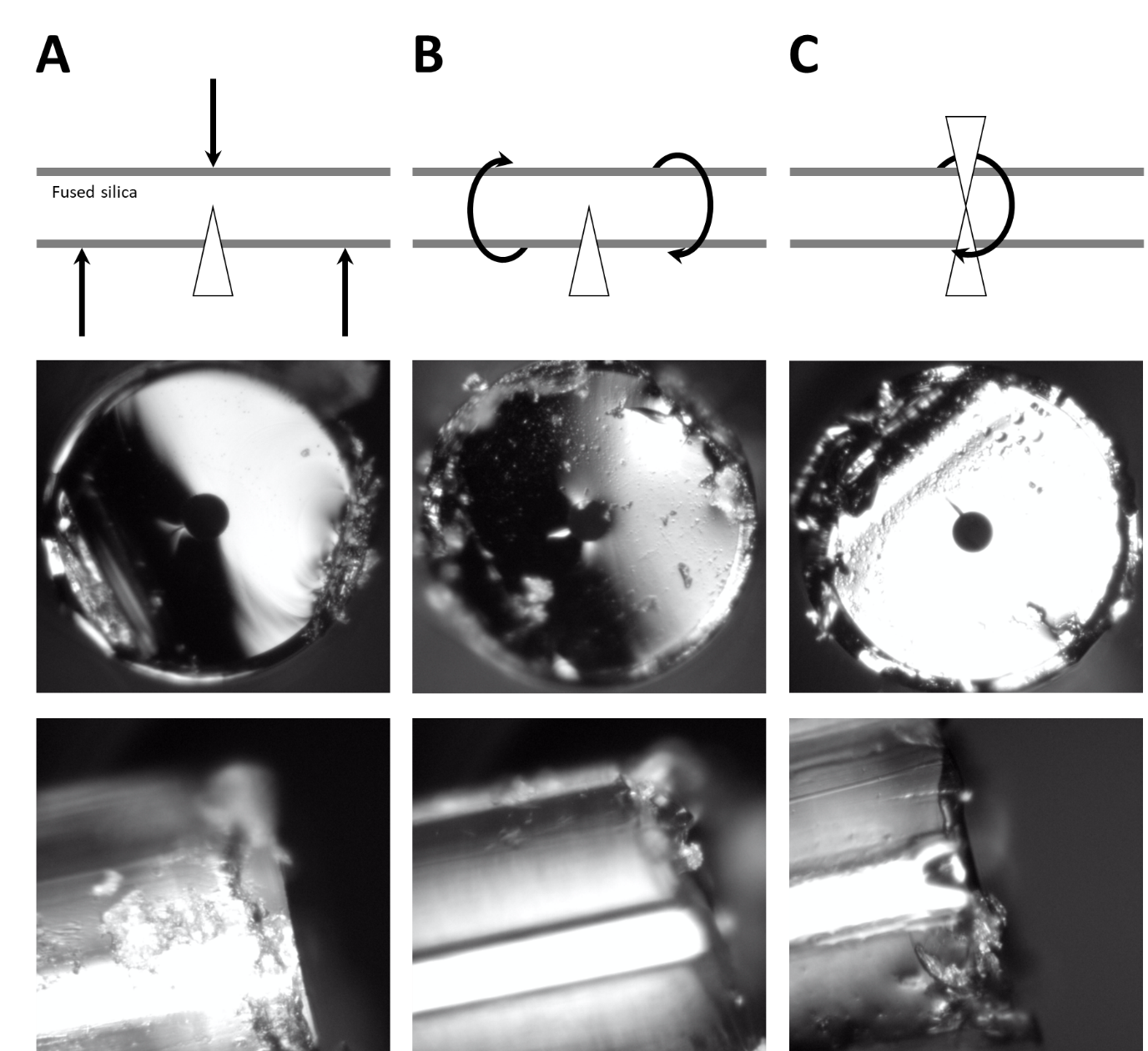


**Figure S1.** Study of the impact of the cutting method of a fused silica capillary; regular cut (**A**), twisted break (**B**) and circular cut (**C**). All cuts were performed using a recent ceramic blade (represented as a triangle in the scheme). The arrows represent the action of the operator fingers on the capillary to provoke the cut. In A and B, a single notch was produced by the ceramic blade. In scheme C, the ceramic blade was turned around the capillary.
